# Nonlymphoma hematological malignancies in systemic lupus erythematosus

**DOI:** 10.1186/ar3998

**Published:** 2012-09-27

**Authors:** M Lu, R Ramsey-Goldman, S Bernatsky, M Petri, S Manzi, MB Urowitz, D Gladman, PR Fortin, E Ginzler, E Yelin, S-C Bae, DJ Wallace, S Jacobsen, MA Dooley, CA Peschken, GS Alarcón, O Nived, L Gottesman, L Criswell, G Sturfelt, L Dreyer, JL Lee, AE Clarke

**Affiliations:** 1 Division of Clinical Epidemiology, McGill University Health Centre, Montreal, QC, Canada; 2 Northwestern University Feinberg School of Medicine, Chicago, IL, USA; 3 Johns Hopkins University School of Medicine, Baltimore, MD, USA; 4 West Penn Allegheny Health System, Pittsburgh, PA, USA; 5 Toronto Western Hospital, Toronto, ON, Canada; 6 Division of Rheumatology, Université de Laval, QC, Canada; 7 State University of New York - Downstate Medical Center, Brooklyn, NY, USA; 8 Division of Rheumatology, University of California San Francisco, San Francisco, CA, USA; 9 The Hospital for Rheumatic Diseases, Hanyang University, Seoul, Korea; 10 Cedars-Sinai Medical Center/David Geffen School of Medicine, University of California Los Angeles, Los Angeles, CA, USA; 11 Rigshospitalet, Copenhagen University Hospital, Copenhagen, Denmark; 12 University of North Carolina at Chapel Hill, Chapel Hill, NC, USA; 13 University of Manitoba, Winnipeg, MB, Canada; 14 The University of Alabama, Birmingham, AL, USA; 15 Lund University Hospital, Lund, Sweden; 16 Rigshospitalet and Gentofte Hospital, Copenhagen University Hospital, Copenhagen, Denmark

## Objective

To describe nonlymphoma hematological malignancies in systemic lupus erythematosus (SLE).

## Methods

An international, multisite (*n *= 28) SLE cohort was linked to regional tumor registries. We examined the types of nonlymphoma hematological cancers occurring after SLE diagnosis, and their demographic characteristics, including sex, race/ethnicity and age at time of cancer diagnosis.

## Results

A total of 15,980 patients were observed for an average of 7.5 person-years. Of these, 90% were female and the majority was Caucasian. Based on age-matched general population cancer rates, the standardized incidence ratio for hematological cancers after SLE onset was 2.9 in females (95% CI = 2.3 to 3.6) and 3.6 in males (95% CI = 2.2 to 5.5). A total of 115 hematological cancers occurred: 82 were lymphoma (75 non-Hodgkin's, seven Hodgkin's), and 33 were nonlymphoma.

Of the 33 nonlymphoma cases, 13 were of lymphoid lineage: multiple myeloma (MM, *n *= 5), plasmacytoma (*n *= 3), B-cell chronic lymphocytic leukemia (B-CLL, *n *= 3), lymphocytic leukemia (*n *= 1), and precursor cell lymphoblastic leukemia (*n *= 1). The remaining 20 cases were of myeloid lineage: myelodysplastic syndrome (MDS, *n *= 7), acute myeloid leukemia (AML, *n *= 7), chronic myeloid leukemia (CML, *n *= 2), and four unspecified leukemias.

All lymphoid malignancies occurred in female Caucasians, except for plasma cell neoplasms, where 4/5 MM cases and 1/3 plasmacytoma cases occurred in blacks (the others being Asian and Caucasian). At the time of MM diagnosis in SLE, the median age was 49 years (range 45 to 57), while for the three plasmacytoma SLE cases the median age was 35 years (range 25 to 62). In the female general population, median age at onset is 70 years for MM [1] and 55 years for plasmacytomas [2]. The median age of SLE subjects at B-CLL onset was 65 years (range 58 to 83), similar to the female general population (74 years).

Of 20 myeloid malignancies, three (15%) occurred in males, and six of the 20 myeloid malignancies (30%) occurred in blacks. All seven AML cases were female, with median age at AML diagnosis of 48 years (range 34 to 72), versus 66 years in the female general population. The seven MDS cases (six females) occurred at a median age of 48 years (range 36 to 59), versus 76 years in the general population. The ages at time of diagnosis for the two CML cases (one female) were similar to the general population median (65 years).

## Conclusion

In our SLE cohort, the most common nonlymphoma hematological malignancies observed were myeloid types (MDS and AML). This is in contrast to the general population, where lymphoid types are three times more common than myeloid [3]. Most (80%) MM cases in our SLE cohort occurred in blacks. Most of our nonlymphoma hematological malignancy cases were younger than general population median age of onset, although this could simply reflect our cohort demographics.
